# Mitochondrial Pharmacology of Dimebon (Latrepirdine) Calls for a New Look at its Possible Therapeutic Potential in Alzheimer’s Disease

**DOI:** 10.14336/AD.2017.1014

**Published:** 2018-08-01

**Authors:** Schamim H Eckert, Janett Gaca, Nathalie Kolesova, Kristina Friedland, Gunter P Eckert, Walter E Muller

**Affiliations:** ^1^Department of Pharmacology, University of Frankfurt/M, Biocenter, D-60438 Frankfurt, Germany; ^2^Deparment of Molecular and Clinical Pharmacy, University of Erlangen, D-91058 Erlangen, Germany; ^3^Department of Nutricional Sciences, University of Giessen, D-35392 Giessen, Germany

**Keywords:** Alzheimer disease, mitochondrial dysfunction, dimebon, latrepirdine, cognitive decline

## Abstract

Dimebon (latrepirdine), an old antihistaminic drug, showed divergent results in two large clinical trials in Alzheimer disease (AD), which according to our review might be related to the specific pharmacological properties of the drug and the different patient populations included in both studies. Out of the many pharmacological effects of Dimebon, improvement of impaired mitochondrial function seeems to be most relevant for the substantial effects on cognition and behaviour reported in one of the studies, as these effects are already present at the low concentrations of dimebon measured in plasma and tissues of patients and experimental animals. Since impaired mitochondrial function seems to be the major driving force for the progression of the clinical symptoms and since most of the clinical benefits of dimebon originate from an effect on the symptomatic deterioration, mitochondrial improvement can also explain the lack of efficacy of this drug in another clinical trial where symptoms of the patiets remained stable for the time of the study. Accordingly, it seems worthwhile to reevaluate the clinical data to proof that clinical response is correlated with high levels of Neuropsychiatric Symptoms as these show a good relationship to the individual speed of symptomatic decline in AD patients related to mitochondrial dysfunction.

Dimebon (latrepirdine) represents an old antihistaminic drug (first generation H1-antagonist), originally developed and clinically used in Russia as an antiallergic drug [1,2] Based on some preclinical studies and findings about robust cognition enhancing properties in a small group of Alzheimer disease (AD) patients, [1] a large placebo controlled phase II trial was carried out in nearly 200 AD patients indicating substantial therapeutic benefit over placebo after 24 weeks not only for cognitive symptoms and for activities of daily living, but also for neuropsychiatric (mainly affective) symptoms [3] Dimebons large effect was driven by an improvement over baseline and even more by the reduction of the typical deterioration of AD symptoms in the placebo group. The substantial therapeutic effects of dimebon remained stable in a continuation phase over additional 6 months. Dimebon’s potential use in geriatric memory disorders was also supported by reports about small cognition improving effects in Huntington disease patients [4], but contrary to the AD trial, the effect was only driven by an improvement over baseline while no change (improvement or deterioration) was seen in the placebo group. This positive effect was not reproduced in a second larger trial where both groups (dimebon and placebo) improved to about the same extent (1.5 MMSE points) with no placebo-verum difference [5]. Similarly, a larger consecutive trial in AD patients failed to show positive effects of dimebon over a similar study time (6 month) and for a similar dimebon dose (20 mg, tid) [6]. Contrary to the initial trial [3] where the placebo group got worst over time (a reduction on the ADAS-cog scale by about 2.0 points) the placebo group in the second trial improved over time by 1.2 ADAS-cog points.

These negative data immediately led to a discontinuation of the clinical development program of dimebon because of lack of activity [6], but also because of lack of clear data about a possible mechanism of action for this drug. Meanwhile much more information about the pharmacological and pharmacokinetic properties of dimebon became available which call for a reconsideration of the clinical data in AD by emphasizing much more carefully possible differences between the patient groups in the two above mentioned trials [7-10].

## Pharmakokinetics and pharmacology of dimebon

### Pharmakokinetics

When the clinical studies mentioned above were carried out, very little was known about the pharmacokinetics of dimebon [11]. Subsequent studies in mice and rats reported dimebon plasma levels at pharmacologically active oral doses between 10 to 50 pmol/ml with about 2 to 3 times higher concentrations for the very short plasma peak around 30 min after administration [12,13]. Elimination was very rapid with most of dimebon excreted after 6 h. Concentrations in mouse and rat brain were about ten times higher compared to the respective plasma levels. Plasma levels reported for AD patients taking dimebon at an oral dose of 20 mg tid were around 10-15 pmol/ml [3,14]. These data agree with a recent study in human volunteers showing peak plasma levels of around 1.3 ng/ml (about 5 pmol/ml) after a single oral dose of 10 mg dimebon [15]. Using the brain/plasma ratios reported for mice or rats therapeutical brain levels of dimebon therefore can be estimated to be around 100 pmol/ml [14].

Bioavailability of oral dimebon seems to be poor as seen in a study comparing oral dosing with transdermal administration due to a large first pass metabolism and shows a large individual variability with up to 20 times differences of AUC values in poor or extensive CYP2D6 metabolizers [15].

### Receptor Pharmacology

As a classical H1-antihistaminic drug dimebon displays high affinity for the H1-receptor with an Ki value around 1 nmol/l [1,12,16]. Contrary to most other old antihistaminics, dimebon does not have anticholinergic (muscarinic receptor antagonistic) properties [12].

Nanomolar target affinity (H1 receptor) agrees with its daily dose as an antiallergic drug (10-20 mg tid) [17] and its plasma concentration in AD patiens when used at the same dose (10-15 nmol/l) [3]. It is also a potent 5-HT7 ligand with an Ki value of 7.0 nmol/l, and also interacts with several 5-HT2, 5-HT6, and alpha2 adrenergic receptor within the relevant concentration range (see above) up to 50 or even 100 nmol/l [12]. Dimebon has also been reported to engage with several other neuronal mechanisms like NMDA and AMPA receptors, Ca-channels, acetylcholinesterase, and glutamate release, but the concentrations required (over 10.000 nmol/l) areclearly outside possible therapeutic brain concentrations [1,13,16,18,19].

Its 5-HT6 receptor antagonistic properties (Ki around 30 nmol/l) [12,20] have been of special interest because of cognition enhancing properties of this group of ligands and their possible use in AD [20,21]. Small cognition improving effects of single doses of dimebon (10-30 mg/kg, ip) have been reported in a rat social recognition test where half maximal occupation of 5-HT6 receptors was observed at a brain concentration of 400 nml/l [20]. This is much higher than the brain concentrations which have been estimated for AD patients (see above). Moreover, in a small study in AD patients, cognitive improvement by a 5-HT6 antagonist was seen relative to baseline and also in respect to the little decline over study time (24 weeks) [21]. Thus, although a contribution of 5-HT6 antagonistic properties of dimebonto, its beneficial effects in AD [3] cannot completely be ruled out, it does not seem to be very likely.

### Cognition improving properties

Because of the complete failure to show any procognitive effect in the second AD trial it is important to review several animal studies reporting improved cognition after dimebon administration. Giorgetti et al. [12] reported improved object recognition behaviour at single oral doses leading to brain concentrations between 1.7 and 170 nmol/L, were maximal effect was already seen at 5 nmol/l. Cognition improving effects were also seen after 31 days of treatment with a ip dose of 3.5 mg/kg in a transgenic mouse model expressing high ß-amyloid levels but not in the non-transgenic littermates [22]. Dimebon also enhanced cognition in rats after lesions of the cholinergic forebrain system [18]. Improved cognition in a hippocampus-dependent learning task was also found in mice after acute (0.5 mg/kg) or repeated (0.1 mg/kg) dosing with dimebon [23]. Dimebon failed to improve learning of young rats in a water maze task following ip doses of 1 mg/kg for 8 days, and also of aged animals with reduced learning abilities [24]. By contrast, dimebon improved working memory in adult and aged monkeys at rather low doses (0.1 mg/kg im), and also in adult animals after impairment with scopolamine [25].

In a mouse model for depression, aged but not young animals showed anhedonic like behaviour (reduction of sucrose preference) [26]. In possible analogy to the beneficial effects of dimebon on Neuropsychiatric symptoms in both AD trials [3,6], treatment of aged (18 months) but not of young (3 months) mice with dimebon for 4 weeks reduced the anhedonic profile [26].

In summary, there is good evidence that dimebon can improve several cognitive functions specifically following impairment as it is the case with many other cognition enhancing compounds, but the data vary substantially with experimental conditions and the cognitive tests used.

## Mitochonodrial pharmacology

When the old antihistaminic drug dimebon was investigated as a cognition enhancer and a novel treatment for AD, the possible mechanism of action became of major interest. Initial reports about neuroprotective and mitochondrial function improving properties specifically following mitochondrial impairment [1,18,27] were confirmed by many subsequent findings in the years following [11]. However, the “novel mitochondrial mechanism of action of dimebon” was also criticized because most of the data published showed mitochondrial protection or improvement only at concentration much over the estimated maximum brain levels for the clinical studies mentioned above [17]. Indeed, positive effects have been observed for neuroprotection against oxidative stress and ß-amyloid toxicity [28-30,] as well as against glutamate neurotoxicity [16], for improvement of autophagy [31-33], and for the inhibition of mPTP (mitochondrial permeability pore) function [34,35] at concentrations higher than 5-10 µmol/l sometimes even more than 100 µmol/l. Interestingly, most of these studies failed to show that these very high concentrations were really needed and that lower concentrations were ineffectice. However, even if very high concentrations were used, the possibility that some of the mitochondrial effect could also take place at lower concentrations still exsists. Indeed. Bharadwaj et al. [7] and Zhang et al. [14] could show improved mitochondrial function at very low concentrations of dimebon which remained stable, even when much higher concentrations were applied.

Accordingly, we will review the mitochondrial pharmacology of dimebon mainly for studies which used concentrations or animal doses in accordance with the known brain or plasma levels discussed above. We include our own mostly published findings where we used a dimebon concentration of 100 nmol/l throughout a large number of different experiments assessing many aspect of impaired mitochondrial function and the beneficial effects of dimebon treatment [36-38]. For further methodological details see our previous publications [39-45]. For these experiments we used HEK cells (HEK_ut_) and a HEK cell line stably transfected with the Swedish APP double mutation (HEK_sw_) [44,45]. While the HEK_sw_ cells served as a model for the detrimental effects of intracellularly generated Aβ on mitochondrial function mirroring the situation in AD brain [46], HEK_ut_ cells were used as a model for the healthy condition. Because of the major role of the brain aging process, treatment of the HEK_ut_ cells with the complex I inhibitor rotenone was used in some experiments as a model for normal brain aging or to mirror the interaction between brain aging and AD pathology [36-39].

### Mitochondrial function

#### Glucose utilisation and OXPHOS activity

Impaired cerebral glucose metabolism arising from memory related brain regions like hippocampus and entorhinal cortex represents an early pathomechanism of Alzheimer’s disease (AD), detectable long before its clinical manifestation [47]. While other mechanisms also play a role (e. g. insulin receptor insensitivity), impaired mitochondrial function seems to be a major cause [49]. According to “the mitochondrial cascade hypothesis of AD”, mitochondrial dysfunction is one of the major mechanism underlying brain aging, mild cognitive impairment (MCI) and late onset Alzheimer’s disease (LOAD)[46,47,48]. The velocity of the decline of mitochondrial function depends on individual genetic predisposition like APOE4 and environmental factors until mitochondrial energy generation falls below a critical threshold. Exceeding this threshold may lead to conditions where mitochondrial dysfunction gets further exaggerated by the combined effects of aging, mildly elevated amyloid-β (Aβ) levels and increased free radical (ROS) make mitochondrial dysfunction a major player within the interface between aging and AD. Furthermore, mitochondrial dysfunction seems to be associated with reduced energy supply and enhanced free radical (ROS) formation finally leading to impaired neuroplasticity including reduced neuritogenesis and neuronal cell loss [50-53]. Thus, improving mitochondrial dysfunction has become an important strategy for the development of drugs to treat the early stages of cognitive decline [45,47,54-59].

Impaired glucose utilisation associated with aging has not only been demonstrated in human brains but also in mouse cortex, hippocampus and somewhat less the cerebellum [60]. Treatment of aged (20 months) but not of young (3 months) mice with dimebon (20 mg/kg ip) 75 min before measuring of glucose uptake with the PET tracer 18-fluoro-deoxyglucose showed significantly enhanced glucose uptake as indicator for a restoration of impaired glucose metabolism [60].

**Figure 1.**
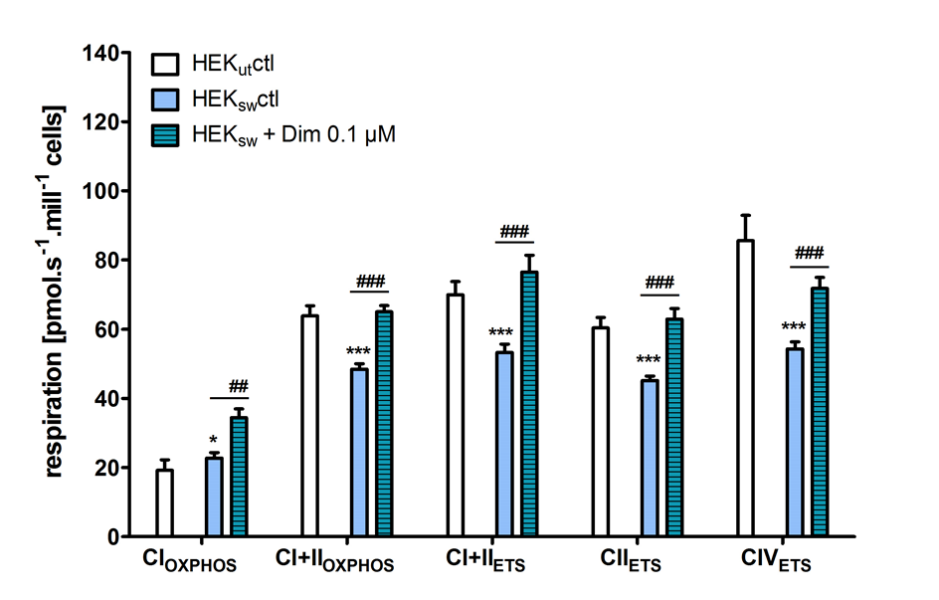
Effects on respiratory activity (adapted and mofified from Eckert et al. [36]) HEK_sw_ cells were incubated for 6 h with dimebon (0.1 µM) and oxygen consumption (respiration [pmolx s^-1^ x mill^-1^ cells]) was measured in different mitochondrial stages by injecting several substrates and inhibitors in an Oxygraph 2k (Innsbruck, Austria). CI_OXPHOS_, CI dependent oxidative phosphorylationdetermined with complex I related substrates glutamate, malate and ADP; CI+II_OXPHOS_, oxidative phosphorylation providing CI and CII substrates by addition of succinate; CI+II_ETS_, non-coupled respiration with CI and CII substrates, is considered as maximum capacity of the ETS by uncoupling with carbonyl cyanide p-(trifluoromethoxy) phenylhydrazone (FCCP, injected stepwise up to 1-1.5 µM); CII_ETS_, non-coupled CII dependent respirationby subsequent inhibition of complex I with rotenone; CIV_ETS_, non-coupled respiration with CIV substrates, applying tetramethylphenylenediamine (TMPD) as an artificial substrate and ascorbate to keep TMPD in the reduced state. Values represent the means ± SEM from n = 6-9 experiments per protocol, Two-way ANOVA with Bonferroni post-tests, *p<0.05, **p<0.01, ***p<0.001.

These findings fit nicely into our findings about effects of dimebon on oxidative phosphorylation activity in HEK cells [36]. Treating HEK control cells with 100 nmol/l dimebon had no effects on OXPHOS activity as measured by high resolution respirometry. The same treatment significantly enhanced OXPHOS actvity in HEK cells with the swedish APP mutation, where OXPHOS was reduced by the overexpression of ß-amyloid to about the same extent (Fig. 1). In both cases, improvement by dimebon was only seen when mitochondrial function was impaired by aging or ß-amyloid overexpression.

To drive complex V as the endpoint of the OXPHOS system to produce ATP, mitochondria utilize glucose derived pyruvate to maintain a proton gradient between the outer and the inner mitochondrial membranes. The resulting mitochondrial membrane potential (MMP) is a sensitive indicator for mitochondrial dysfunction where it is usually reduced. Treating mouse primary neurons or SY5Y neuroblastoma cells with dimebon at low concentrations (1-10 nmol/l) enhanced mitochondrial membrane potential and ATP production [14]. Under stress situations (elevated intracellular calcium, serum depreviation) similar dimebon concentrations protected the cells against the decrease of mitochonsrial membrane potential and led to better survival (reduced apaptosis) [14].

**Figure 2.**
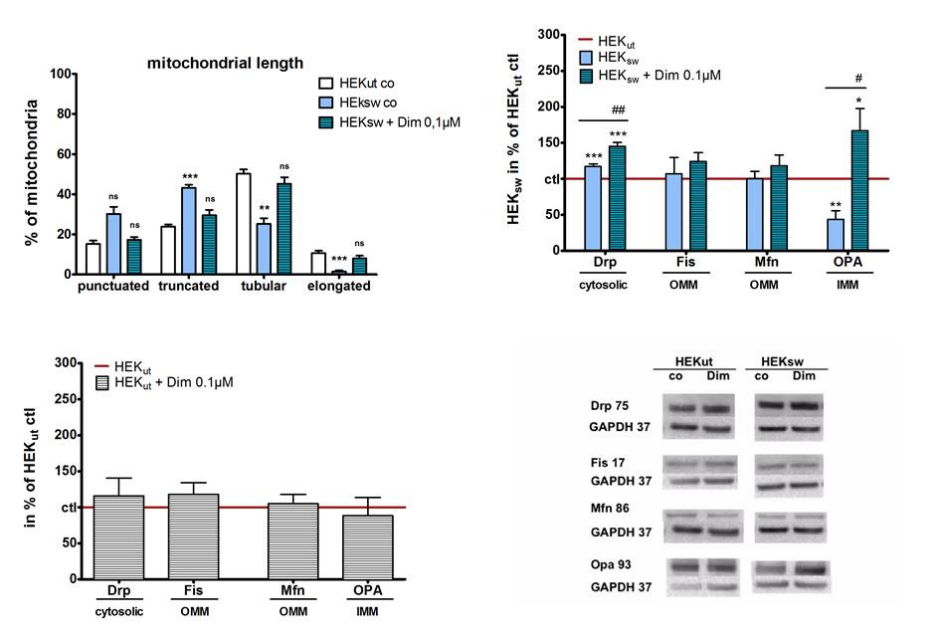
Effects on mitochondrial morphology (adapted and modified from Eckert et al. [36] Müller et al. (37), Eckert (38)) HEK-cells harboring the Swedish mutation in the APP gene (HEKsw) and control cells (HEKut) cells were incubated with dimebon (0.1 µM) for 6 h. (**A**) For the determination of mitochondrial length, organells were labeled with Mito Tracker CMXRos, fixed with PFA. Mitochondrial lengths were quantified using Image J and classified in punctuated, truncated, tubular, and elongated mitochondria. Data represent the means ± SEM with at least 100 measured mitochondria per experiment, n = 8-9, Two-way ANOVA with Bonferroni post-tests, **p<0.01, ***p<0.001. (**B, C**) Effects of dimebon on expression levels of fission and fusion marker. Marker proteins for fission dynamin related protein1 (Drp) and fission 1 related protein (Fis), as well as markers for mitochondrial fusion protein 1 (Mfn) and optic atrophie-1 (Opa) were measured using Western Blot analysis, after electrophoretic separation and using specific antibodies. Cellular location of the proteins in the cytosolic fraction as well as in inner (IMM) and outer (OMM) mitochondrial membranes is indicated. Data were normalized to HEKut (100%) and represent the means ± SEM, n = 8-9, Two-way ANOVA with Bonferroni post-tests, *p<0,05, **p<0,01, ***p<0,001 vs. ctl; #p<0,05, ##p<0,01 vs. HEKsw. (**D**) Representative Western Blots.

Mitochondria are abundant in synaptic terminals and ATP production by mitochondria is crucial for synaptic function. Impaired mitochondrial function associated with reduced ATP supply lead to synaptic dysfunction, reduced neuronal and synaptic outgrowth and finally apoptosis [47,48,50,61]. Many drugs which improve mitochondrial function enhance neuronal survival, improve neurite outgrowth and neuronal proliferation [39-41,53,62]. Similarly, mitochondrial improvement by dimebon (up to 100 nmol/) has been associated with enhanced neurite outgrowth in cortical and hippocampal primary cells and cortical neurons [11,63]. It also enhanced neurite outgrowth in primary cortical cells [64].

The inner mitochondrial membrane harbors the proteins of the electron transport system (ETS), and its integrity is crucial for the respiratory complex (OXPHOS) activity driving ATP production [65]. Since the majority of mitochondrial proteins are nuclear encoded and synthesized in cytosolic ribosomes, import into mitochondria is mediated by various translocases of the outer mitochondrial membrane (TOMM) and translocases of the inner mitochondrial membranes (TIMM). Contrary to the OMM, the IMM requires two translocases for import of precursors into the matrix, TIMM 23 and TIMM 22 [66,67]. TOMM 22 are part of the membrane-embedded core components that are forming the general insertion pore (68,69,70) anchored to the outer membrane and mediating the initial steps for the import of preproteins. TIMM 50 functions as a receptor and component of TIMM 23. It guides the precursors from intermembrane space to the translocation pore TIMM 23.

**Figure 3.**
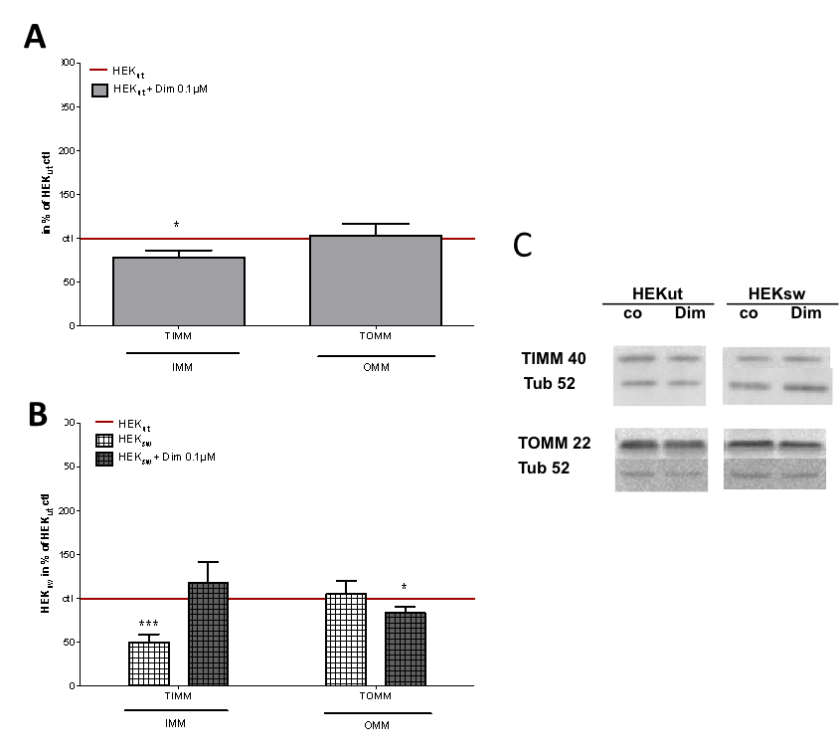
Effects on mitochondrial membrane composition (adapted and modified from Müller et al. [37], Eckert [38]) Cells were incubated with 0,1 μM dimebon (Dim) for 6 h. (**A**) In HEK control cells (HEKut) and (**B**) HEK-cells harboring the Swedish mutation in the APP gene (HEKsw), marker proteins for the inner (IMM) and the outer mitochondrial membrane (OMM), were measured in total homogenates, using Western Blot analysis after electrophoretic separation and using specific antibodies against translocator proteins of the inner (TIMM50) and outer (TOMM22) mitochondrial membrane, respectively. Data were normalized to HEKut (100% in A) and HEKsw (100% in B), respectively. Data represent the means ± SEM, n = 6, Two-way ANOVA with Bonferroni post-tests, *p<0,05, ***p<0,001. (**C**) Representative Western Blots.

**Figure 4.**
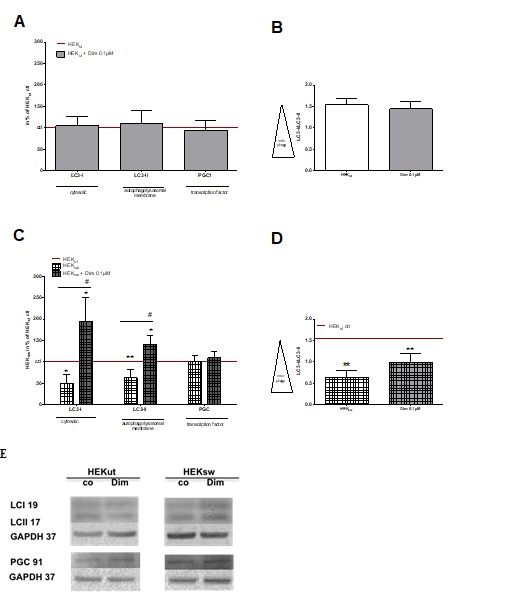
Effects on mitophagy (adapted and modified from Müller et al.[37], Eckert [38]) (**A**) Control cells (HEKut) and (**C**) HEK-cells harboring the Swedish mutation in the APP gene (HEKsw) an were incubated with 0,1 µM dimebon (Dim) for 6 h. Autophagy marker proteins for the cytosol (LC3-I) and autophagosomal membranes (LC3-II) as well as the transcription marker peroxisome proliferation-activated receptor gamma coactivator 1-alpha (PGC), were measured using western blot analysis after electrophoretic separation and using specific antibodies in total cellular homogenates. (**B & D**) A low LC3-I/LC3-II ratio indicates high degree of mitophagy. Data were normalized to HEKut (100%) and represent the means ± SEM, n = 8-9, Two-way ANOVA with Bonferroni post-tests, *p<0,05, **p<0,01 vs. HEKut ctr; ^#^p<0,05 vs. HEKsw ctl. (**E**) Representative Western Blots.

To get information whether the integrity of the inner membrane is involved, we investigated expression levels of TIMM50 in our cell system. HEKsw cells show a large reduction of TIMM expression levels (Fig. 3B). Dimebon treatment increases TIMM expression back to control levels (Fig. 3B) which parallels the restored OXPHOS capacity (Fig 1). Incubation of control cells with dimebon only slightly decreases expression levels of TIMM (Fig. 3A) which seems to parallel that OXPHOS activity is not affected under similar conditions [36]. The outer membrane component OMM is not altered by the transgen (HEKsw cells) or by dimebon treatment of the HEKut cells but is slightly decreased in the HEKsw cells by the addition of dimebon (Fig 3A & B). This minor effect of dimebon on mitochondrial membrane compositon in HEWsw cells seems to go in parallel with as small increase of mitochondrial mass only [36]. It is further supported by our findings that PGC-1α (peroxisome-proliferator-activated receptor γ co-activator-1α) is not altered (Fig. 4A & C). PGC-1α is a transcriptional co-activator that serves a master regulator of mitochondrial biogenesis and of antoxidant defense mechanisms [42,43,71,72].

### Mitochondrial quality control

Mitochondrial dysfunction as it occurs in aging and many neurodegenerative diseases like AD usually takes years or even decades before symptoms arise, since it only gets functional relevant, when the rate of damage exceeds the rate of continual repair by the mitochondrial quality control system (QC). Mitochondrial dynamics and autophagy are integral part of this QC system [73,74] which seems to be substantially disturbed in AD [75]. Many data suggest that the beneficial effects of dimebon on mitochondrial function are at least in part related to beneficial effects on reduced qualitity control.

#### Mitochondrial dynamics

Mitochondrial dynamics, meaning the ability of mitochondria to undergo changes in size and form [76]are gaining more and more attention as an important factor regulating mitochondrial function and as mechanism of mitochondrial quality control. Even if reports are sometimes controversial, in most cases mitochondrial fragmentation is accompanied by reduced mitochondrial function and vice versa [77-81]. Accordingly, shorter mitochondria are energetically unfavorable. We have previously used confocal microscopy of fixed mitochondria as a very reliable method to analyze mitochondrial dynamics in many situations of impaired mitochondrial function and to demonstrate beneficial effects of several drugs on the fission and fusion balance [36,39-41,55].

As reported previously, the pronounced Aß production in HEK_sw_ cells [36,55] goes parallel with substantial changes of mitochondrial dynamics, shifting mitochondrial size to smaller (fission) mitochondria (punctuated form) (Fig. 2 A). Treating these cells with dimebon restores changes of mitochondrial morphology (Fig. 2 A) nearly back to control values.

Mitochondrial fission and fusion balance is regulated by the interaction of mainly two proteins: the cytosolic GTPase dynamin-related protein 1 (Drp1) and an outer mitochondrial membrane anchored protein, mitochondrial fission protein 1 (Fis1). Fusion processes are chiefly regulated by the two GTP-ase isoforms: mitofusin 1 and 2 (Mfn1 and Mfn2), as well as optic atrophy type 1 (OPA1). Fission and fusion events are very frequent, take place within a few minutes [73,81,82] and exchange matrix and inner and outer membrane proteins under are carefully balanced conditions [82,83]. While fused mitochondria seem to be the energetically more relevant form, at least one function of fission is the sorting out of deficient mitochondrial fragments and to activate autophagy of the respective mitochondrion (mitophagy) [73,81].

After our initial findings indicating that dimebon restores changes of mitochondrial morphology in Aβ overexpressing HEK cells [36] independent of ROS, we examined whether changes in morphology were related to altered expression of key proteins involved in mitochondrial dynamics. We detected substantial differences between controls and HEKsw cells, supporting previous findings about pronounced effects of Aß on mitochondrial dynamics (Fig. 2 B). While Fis1 and Mfn1, both located in the outer mitochondrial membrane, are unaltered in our HEKsw model, levels of Drp are upregulated, whereas levels of OPA are distinctively downregulated (Fig. 2B). To test the effect of Dimebon on mitochondrial dynamics, cells were incubated with Dimebon (100 nM) for 6 h. While dimebon has no effect on levels of fission and fusion marker in HEK control cells (Fig. 2C), it shows broad efficacy in the AD cell model (Fig. 2B). Dimebon treatment is further enhancing expression levels of Drp and in case of OPA1, Dimebon ameliorates the disease-specific deficit. Our data suggest that changes in mitochondrial morphology observed in Aβ overexpressing HEK cells and the normalization by dimebon [36] were related to the altered expression pattern of mitochondrial fission and fusion proteins (Fig. 2B).

#### Autophagy

Autophagy (or mitophagy in case of mitochondria) represents an important quality control system to degrade damaged proteins and organelles and to reintroduce their constituents back to the cytosol as nutrients for renewal [84,85]. Moreover, dysfunctional mitochondria can be selectively removed by mitophagy. Dysregulation of mitophagy is implicated in the development of neurodegenerative disease. If damaged mitochondria are not degraded, their increased reactive oxygen species (ROS) production can damage the cell [84,86,87]. Dysregulation of mitophagy with the risk of elevated ROS production has been implicated in the development of neurodegenerative diseases in general and with the neuropathology of AD in specific [84].

Alterations in mitochondrial morphology, dynamics, as well as deficits in the electron transport capacity of the respiratory chain seem to be mutually connected to changes in the quality control and turnover of mitochondria [36,41,87]. Accordingly, our HEKsw cells show decreased levels of proteins involved in autophagic processes. LC3-I and LC3-II, are prominent indicators for autophagy (Fig. 4C). The conversion of LC3-I to LC3-II is a specific marker for autophagic activity. Reduced conversion to LC3-II indicates decreased formation of autophagosomes [88]. Treatment of HEK control cells with dimebon does not influence expression levels of autophagic marker (LC3-I and LC3-II). By contrast, Aß-related decreases in HEK_sw_ cellsof LC3-I and LC3-II, are compensated by dimebon treatment at nanomolar concentrations (Fig. 3C) suggesting restoration of impaired mitophagy [88,89].

Several other data suggest autophagy stimulating properties of dimebon in different cell and animal models [7,10,32,33]. Steele et al. [33,33] measured the formation of autophagosomes by counting LC3 punctae in HeLa cells and LC3-II levels of mouse N2a neuroblastoma cells after 3 or 6 h acute treatment with dimebon. However, very high dimebon concentrations (50-100 µmol/l) were used in this study. Nevertheless, the findings parallel our experiments (6 h treatment with 100 nmol/l dimebon in HEKsw cells with increased LC3-I and LC3-II levels. In cells and mouse brain, dimebon was able to decrease levels of α-synuclein by stimulating autophagy [32,33]. Enhanced autophagy was also seen in a yeast model with first effects of dimebon at a concentration of 250 nmol/l [7]. α-Synuclein is a protein which is related to neurodegenerative diseases especially Parkinsonism while Ɣ-synuclein seems to be associated with the neurodegenerative disease ALS (amyotrophic lateral sclerosis).

**Figure 5.**
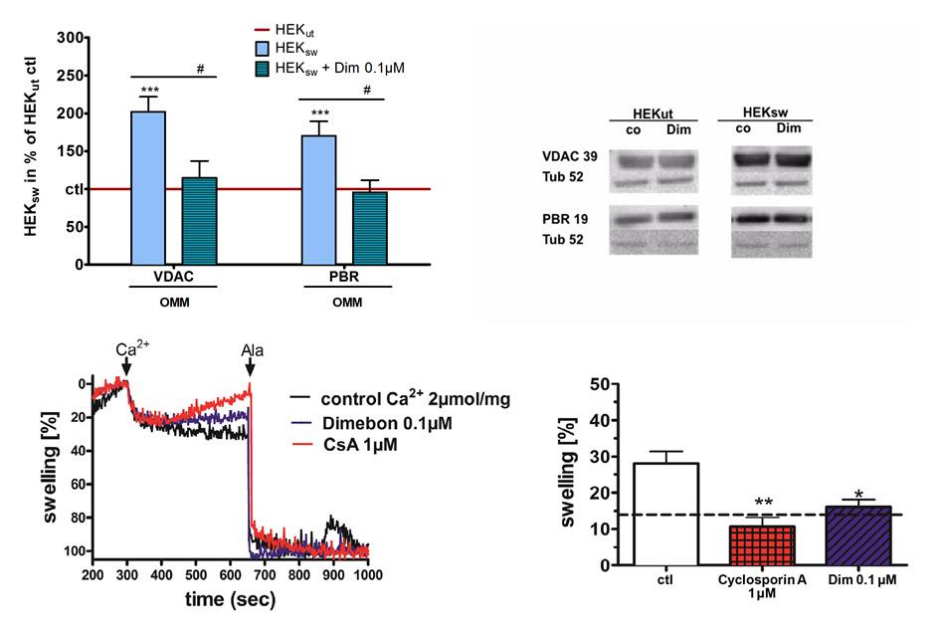
Effects on structure and function of the mitochondrial permeability transition pore (mPTP) (adapted and modified from Müller et al. [36]) (**A**) HEK_sw_cells were incubated with 0,1 µM dimebon (Dim) for 6 h, mPTP marker proteins of the outer mitochondrial membranes (OMM), voltage-depended anion channel (VDAC) and peripheral benzodiazepine receptor (PBR), were examined using western blot analysis after electrophoretic separation and using specific antibodies in total homogenates. Data were normalized to HEKut (100%) and represent the means ± SEM, n = 8-9, Two-way ANOVA with Bonferroni post-tests, **p<0,01, *p<0,05, ***p<0,001 vs. HEKut ctl; ^#^p<0,05 vs. HEKsw ctl. (**B**) Representative Western Blots. In HEKsw cells, dimebon dramatically restored the increased expression levels of these mPTP markers (Fig 5 A). (**C**) Exemplary graph of a measurement of light scattering which is equivalent to mitochondrial swelling; Ca2+: inductor of physiological extent of mitochondrial swelling; Ala: Alamethicin [3.2 mg/mL], inductor of maximal mitochondrial swelling. (**D**) Swelling of isolated mitochondria from female NMRI mice challenged with calcium (Ca^2+^, 2 mmol/mg protein) and simultaneously incubated with cyclosporin A, a known inhibitor of mitochondrial swelling (CsA, 1 µM) and dimebon (0.1 µM; statistics were calculated against calcium insult; (; n=5-8; mean ± SEM; p*<0.05; p**<0.01; p***<0.001

In a transgenic mouse model of ALS overexpressing this protein, dimebon treatment (3 or 6 mg/kg) for several months not ony reduced Ɣ-synuclein load but also motor performance [90]. Since an age-dependent decrease of autophagy has been identified as a feature for the progression of AD pathology [84-86,91], the findings that dimebon improves autophagy (mitophagy) in several disease models certainly could be relevant and should be further focused on.

### The Mitochondrial Permeability Transition Pore as possible target

The mPTP represents a dynamic multiprotein complex which spans the inner and outer mitochondrial membranes at special contact sites [92]. Although, the structure of the mPTP is not yet fully elucidated, there are several identified components or modulators of the mPTP. The most common proposed structure of mPTP includes the voltage-dependent anion channel (VDAC) and the 18kDa translocator protein (TSPO; formerly known as the peripheral benzodiazepine receptor), in the outer membrane, the adenine nucleotide translocator (ANT) in the inner membrane, cyclophilin D (Cyc D) from the matrix and possibly other proteins such as creatine kinase (CK) from the intermembrane space, hexokinase (HK) at the outer surface of the outer membrane, and pro-apoptotic proteins of the Bcl-2 family such as Bax. Opening of the mPTP is followed by a sudden increase of permeability of mitochondrial membranes, which allows solutes up to 1500 Dalton to equilibrate between mitochondrial matrix and cytosol. This leads to uncoupling of oxidative phosphorylation system, mitochondrial matrix swelling, dissipation of MMP, increased ROS production, and releases of apoptotic proteins (see above) [92-94]. Opening of mPTP may play a causative role in mitochondrial fragmentation, depolarization of the mitochondrial membrane potential, ATP depletion and finally apoptosis. Supporting our data, inhibition of mPTP in other disease models already showed both, reduction in expression of fission proteins and increase in expression of fusion proteins and impaired fission and fusion balance [41,95,97].

Numerous effectors can open the mPTP in particular calcium ions, reactive oxygen species, and amyloid-ß and on the other hand many endogenous and exogenous inhibitors of mPTP have been described including high negative potential, low matrix pH, ADP, magnesium and strontium, and the immunosuppresive drug cyclosporine A [41, 96-98].

#### Dimebon restores expression levels of mPTP associated proteins back to control levels

As mitochondria are the main source of ROS production, they are especially prone to ROS damage. Such damage can induce opening of the mitochondrial permeability transition pore (mPTP), which leads to mitochondrial swelling and cytochrome c release and can therefore initiate apoptosis upon its opening [65]. To investigate possible alterations of core components of the mPTP, we determined expression levels of voltage dependent anion channel (VDAC) and peripheral benzodiazepine receptor (PBR; also called translocator protein; TSPO). Both proteins are located in the outher mitochondrial membrane. In HEKsw cells expression levels of both, VDAC and PBR were strongly increased (Fig. 5A). These findings fit recent data from our lab, that apoptosis occurs more often in HEKsw cells compared to controls [55]. Dimebon elevates both core components of mPTP in HEK control cells only to some extent (data not shown), while in our disease cell model, levels of both mPTP components are dramatically elevated compared to the untreated control (Fig. 5A), which in consequence can cause stronger vulnerability to stressors (Fig. 5B). Dimebon is completely restoring the VDAC and PBR expression levels back to untreated control levels (Fig. 5A).

#### Dimebon inhibits mPTP function

Induction of mPTP opening leads to a nonspecific high permeability for different agents, to a collapse of MMP and loss of ATP. This finally ends in the rupture of the OMM and release of proapoptotic intermembrane proteins into the cytosol as, cytochrome c [94]. Cyclosporin A inhibits mPTP trough interaction with cylophilin D [98]. This was confirmed in our experiments [40,41] where cyclosporine largely inhibited mPTP opening by calcium. Dimebon showed a similar also slightly smaller effect at 100 nmol/l (Fig. 5). Similar effects have previously been reported for dimebon although much higher concentrations of the drug (200 or 50 µmol/l) were used [34,35].

Even though there are still multiple models and viewpoints regarding mPTP and its components, the prevention of mPTP opening has been shown to provide neuroprotection in different paradigms [92]. As mPTP opening regulates many mitochondrial functions (OXPHOS activity, dynamics, quality control) the effects of dimebon on mPTP function could be relevant for most of dimebon’s effects at the mitochondrial level.

## Mitochondrial improvement by dimebon, Alzheimer treatment beyond ß-amyloid?

When dimebon was investigated as a cognition enhancer and possible AD treatment based on initial positive results in AD patients [1,2,3,27] its novel mitochondrial mechanism of action was seen with major interest as more and more negative clinical findings became published in respect to Aß directed possible AD treatment strategies including inhibitors of Aß aggregation and production from the precursor protein as well as antibodies or vaccination to remove it out of the brain. [48, 53, 99, 100]. Even if all seemed to remove Aβ to some extent, all strategies failed to improve the symptoms of dementia, some of the treatments made dementia even worse [47,99,100]. Moreover, recent advances in imaging techniques confirm that many patients die quite old with a large Aβ plaque load without showing symptoms of dementia and that accumulation of Aβ containing plaques already reaches its maximum decades before first symptom of cognitive impairment or even dementia develop [101-103]. Thus, it became quite clear that the simple Aβ cascade hypothesis has failed, especially as basis for the development of new AD specific drugs. Accordingly, other aspects of AD pathology, more closely related to the clinical symptoms of the disease (Alzheimer’s dementia) are currently investigated as targets for therapeutic improvement likesynaptic deficits, which do correlate with the presence of the disease.

One alternative concept to explain AD relates to the mitochondrial cascade hypothesis of AD which assumes mitochondrial dysfunction and elevated stress as one major pathomechanism underlaying the whole spectrum of age-associated cognitive disorders from rather subjective cognitive complaints at older age over mild cognitive impairment (MCI) to AD and VD (vascular dementia) [47,5]) and the suggestion that drugs improving mitochondrial dysfunction could serve as possible treatment for AD [48,50,52,54,55,58,104]. Accordingly, when the first data about positive effects of dimebon in AD got published [1,3] the “novel mitochondrial mechanism of action” was enthusiasticaly seen, but on the other site rapidly dropped once negative data were reported [17]. However, since major differences came up regarding design and patient’s characteristic of the clincal studies and regarding the extensive data about effects of dimebon at the mitochondrial level as reviewed above, a critical discussion of the clinical studies seems justified. The first open study with 12 AD patients who were treated with dimebon for 4 weeks only provided a first signal [1]. The follwing phase II study gave substantial evidence for beneficial effect of dimebon in AD. Even planned as a phase II study it comes close in design and size to a phase III study [3]. In this randomized double-blind placebo controlled study, 183 patients were treated for 26 weeks with dimebon (20 mg t.i.d.) or placebo. Outcome measures included assessment of cogniton (ADA-cog, MMSE), daily life function (ADCS-ADL) and behaviour or neuropsychiatric symptoms (NPI). After 26 weeks, dimebon was significantly superior over placebo with substantial placebo-verum differences (4.0 points ADAS-cog; 2.2 points MMSE; 3.4 points ADCS-ADL; 3,6 points NPI). On all measures, a substantial decline of function was seen at endpoint in the placebo group as a sign of the typical deterioration of the disease. On all measures, the improvement by dimebon seen already after 3 months further increased until week 26 or at least remaind stable. For the first 12 weeks, placebo remained rather stable or even increased slightly (MMSE) but than dropped under baseline.

While this placebo pattern has also been typicallyseen in pivotal trials with donepezil and other acetylcholine esterase inhibitors [105], the improvement seen for these drugs after several months did not remain stable but declined over time rather parallel to the decline of the placebo patients. The stable improvement by dimebon by continuos decline under placebo has been interpreted as possible indication of a disease-modifying effect of dimebon. This conclusion was further supported by a 26 weeks double-blind continuation phase of the trial with about 130 of the originally 180 patients [3]. While the placebo patients showed further deterientation on the ADAS-cog scale the dimebon group remained nearly stable leading to a much higher placebo-verum difference at week 52 (6.9. points). Continuous improvement by dimebon until week 52 was also seenin the presence of further decline in the placebo patients for all other scales. About 100 patients of both groups were further treated for another 26 weeks in an open design with dimebon. The originally dimebon treated patients still showed a substantial improvement [11] while the original placebo patients improved significantly under dimebon relative to the 52 weeks baseline. Taking together, these findings strongly suggested that a large part of dimebons benefical effect were related to a slowing down ofthe decline over time of the cognitive and behavioral symptoms of the AD patients.

Following this study showing substantial benefits of dimebon treatment in AD, several other phase III trials were initiated, only one was finished [6,17]. This was a randomized placebo-controlled doube-blind trial where nearly 600 patients were treated for 26 weeks with placebo or either 5 or 20 mg dimebon t.i.d. Clinical efficacy was evaluated using the same rating scales already used in the Doody et al [3] trial. The data of this trial have not been published in detail [6,17]. Plasma levels have not been reported, which would be relevant regading the large first-pass metabolism of the drug and the use of a different dimebon formulation. To make a long story short, no significant improvement over placebo was seen for both doses of dimebon on all measures. None of the measures showed a significant decline over the 26-week period. On the MMSE the dimebon patients improved by 0.7 points but the placebo patients improved even by 1.2 points! This is rather strange and raises questions about the inclusion criteria of the patients. At least it suggests fundamental differences between the patient characeristics in both trials. The decline of symptomatology seen in the first trial over the 26 weeks period in the placebo group was very much within the range reported for AD patients in many epidemiological studies and typical for these patients. Showing no decline or even an improvement within 26 weeks is rather atypical for this disease. Thus, if dimebons benefical effects are related to slowing down the deterioration of symptomatology very little benefit can be expected in a patient population which gets better on placebo.

The problem which arises is the question if we can predict deterioration of symptomatology in AD patients. The patients in the Doody et al. [3] study showed a substantial level of Neuropsychiatric symptoms (NPS) at baseline which significantly improved by dimebon treatment. NPS are typical symptoms of dementia and are present in most patients with AD or VD. The prevalence of NPS in different populations of patients with dementia is around 80-90% [106-112]. Thus, the presence of NPS is rather the norm than the exception. One major aspect oft the presence of NPS in dementia seems to be a faster cognitive decline in these patients [109,111,113-115]. Even if the neurobiology of NPS in dementia is not yet completely understood, patients with NPS or symptoms typical for NPS seem to have pronounced mitochondrial dysfunction beside other neurobiological deficits [116-120]. Thus it seems that in the Doody et al. [3] trial typical AD patients with the presence of NPS and a decline of cogniton over time were included which seems rather not the case for the second dimebon trial [6]. It would be easy to go back to the datasets to see if the patients with high NPS values are the one who respond to dimebon or show decline in the placebo group. This scenario has been shown in a large clinical trial with the ginkgo special extract Egb761 [115] which improves impairend cognition over the whole spectrum of age-associated cognitive disorders from MCI to AD or even VD by enhancing mitochondrial function [53]. In this trial,a better response of cognitive symptoms to EGb761^®^and a faster cognitive decline has been reported for those patients with high levels of NPS [120].

## Final conclusion

Improvement of impaired mitochondrial function seems to be the most relevant pharmacological property of dimebon already present at the low plasma and tissue levels meassured in patients with AD and therefore seems to be the basis of its positive effects on cognition and behaviour in AD patients. Since impaired mitochondrial function seems to be the most relant driving force for the progression of the clinical symptoms of the disease and most of the clinical benefits of dimebon originate from an effect on the symptomatic deterioration, this mechanism of action also can explain the lack of efficacy of this drug in patients whose symptoms remain stable for the time of the clinical study. It seems worthwhile to reevaluate the clinical data to proof that clinical response is correlated with high levels of NPS as these show a good relationship to the individual speed of symptomatic decline in AD patients related to mitochondrial dysfunction.
